# Unmasking Acute Lymphoblastic Leukemia in a Chiropractic Patient With Musculoskeletal Complaints

**DOI:** 10.7759/cureus.40675

**Published:** 2023-06-20

**Authors:** Tze Kwan Sharon Mok, Benjamin Kah Chun Cheong, Kevin Hsu Kai Huang

**Affiliations:** 1 Chiropractic, New York Chiropractic and Physiotherapy Centre, New York Medical Group, Hong Kong, CHN

**Keywords:** philadelphia chromosome-positive acute lymphoblastic leukemia, low back pain, chiropractor, chiropractic, acute lymphoblastic leukemia

## Abstract

A 67-year-old woman presented to a chiropractor with a four-week history of neck and low back pain, lower extremity paresthesia, profound fatigue, and cutaneous pallor. Previous cervical radiographs had revealed multilevel degenerative spondylosis. However, abnormal hematological indices, including severe thrombocytopenia and anemia, prompted concerns of an underlying hematopoietic malignancy. Interdisciplinary collaboration facilitated expedient hematological assessment, confirming acute lymphoblastic leukemia (ALL), as evidenced by lymphoblasts in a peripheral blood smear and bone marrow biopsy. Karyotyping detected a Philadelphia chromosomal mutation; the patient therefore received oral targeted tyrosine kinase inhibition coupled with serial intrathecal chemotherapy. Complete remission was achieved. However, sensorimotor symptoms persisted due to herpetic neuralgia secondary to immunosuppression. This complex case underscores the role of chiropractors as primary contact clinicians in identifying sinister pathologies underlying musculoskeletal complaints via judicious history-taking, physical evaluation, and interpretation of investigational findings. Interprofessional collaboration is pivotal in formulating an effective therapeutic strategy to improve the prognosis of patients with this disease.

## Introduction

Acute lymphoblastic leukemia (ALL) primarily affects adults aged >65 years and children aged <6 years. It is a rare pathology with a global incidence of 1-5 cases per 100,000 people per year [1] and accounts for approximately 20% of adult leukemia cases [2]. The clinical presentation of ALL varies, with fatigue, fever, arthralgia, bleeding, and bone pain being common manifestations of the disease [1]. Musculoskeletal symptoms are less common in adults with ALL. However, central nervous system (CNS) involvement is occasionally observed [3]. The early detection and diagnosis of ALL are critical for optimal patient outcomes, as prompt initiation of treatment has been shown to result in improved survival rates [4].

Patients with ALL have been reported as presenting to the emergency department with lower back pain [1,3,5,6]. However, searches of PubMed, Google Scholar, and Scopus databases found no previous case report that described a patient with ALL presenting with back pain at a chiropractic clinic, highlighting the novelty of this study in the chiropractic literature. Chiropractors, as primary care providers who routinely evaluate musculoskeletal complaints, may encounter patients presenting with symptoms that could be indicative of an underlying hematological disorder like ALL [7,8]. Lower back pain is a common complaint among patients seeking chiropractic care. Although this pain can predominantly be attributed to musculoskeletal causes, it can also be a symptom of severe diseases such as ALL [9]. In such cases, chiropractors play a crucial role in detecting potential red flags, conducting thorough physical examinations, and ordering relevant diagnostic tests for further investigation [10,11]. Timely referrals to the appropriate specialists, such as hematologists, may facilitate early diagnosis and intervention, thus improving patient prognosis [12].

This case report highlights the vital role of chiropractors in identifying hematological disorders such as ALL in patients presenting with lower back pain. Through a multidisciplinary approach and collaboration, patients with complex presentations can receive appropriate care to address their musculoskeletal and systemic health needs.

## Case presentation

**Figure 1:**
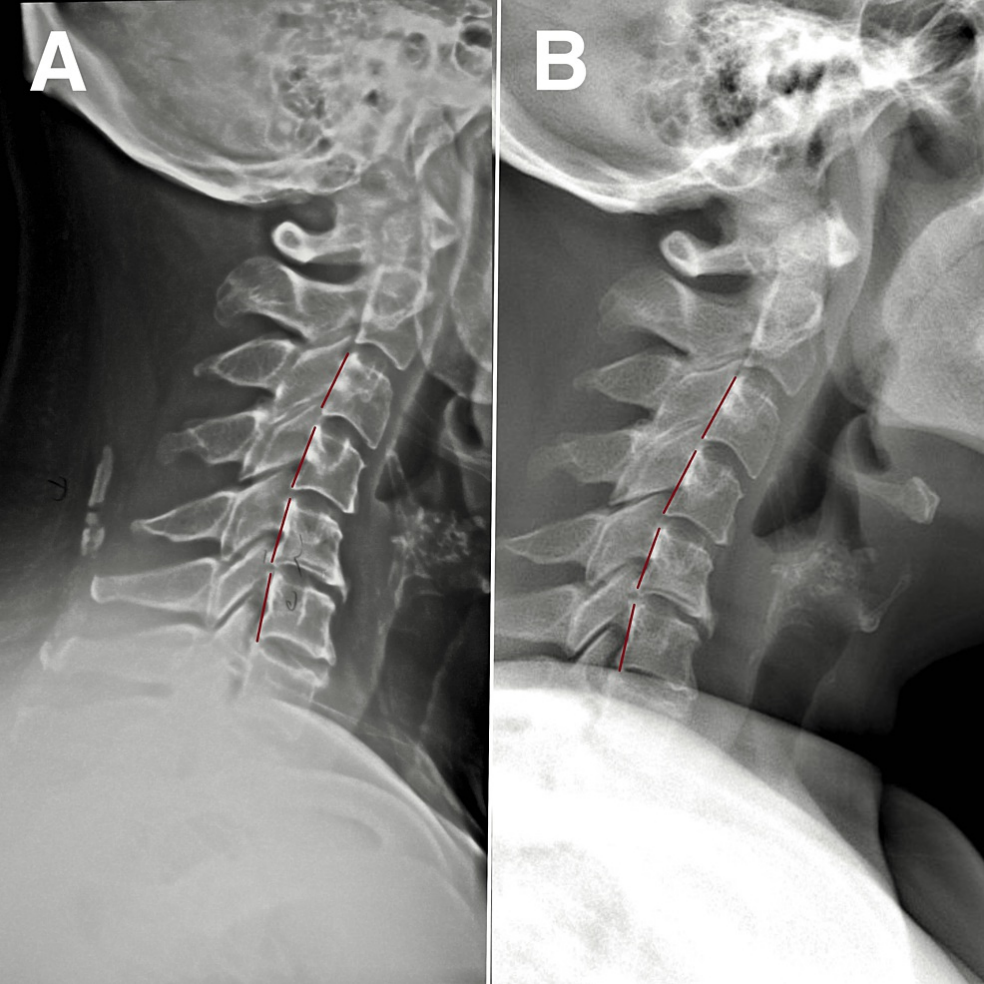
Cervical radiographs (A) Lateral cervical radiograph taken in 2017 showed moderate spondylosis and reversed cervical lordosis. (B) Lateral cervical radiograph taken in 2020 showed worsening of cervical lordosis (as indicated by the red lines).

In March 2022, a 67-year-old woman presented to a chiropractor with mild neck and back pain, numbness and tenderness of the lower extremities, and persistent fatigue, which she had been experiencing for four weeks. The patient had a five-year history of chronic neck and back pain. Her symptoms have been managed well by both physiotherapy and acupuncture. Radiographs taken in 2017 had identified moderate cervical spondylosis (Figure 1A), and follow-up cervical radiographs in 2020 confirmed worsening of cervical lordosis (Figure 1B). During the initial assessment, it was noted that the patient had a pale complexion and darkened facial features. This pallor was more noticeable in the face, lips, and inside the mouth. The nail beds appeared paler white, and the lips were bluish in color. The chiropractor was concerned about the presence of an underlying medical condition and therefore performed a focused physical examination and ordered blood tests for further investigation. The blood tests revealed severe thrombocytopenia with a platelet count of 6 x 10^9/L (normal range: 150-400 x 10^9/L) and anemia with a hemoglobin level of 4.9 g/dL (normal range in females: 12-15.5 g/dL). As the abnormal blood test results indicated a possible hematological disorder, the chiropractor immediately contacted a hematologist for further evaluation as the management was beyond the chiropractor's scope of practice. The patient was referred and saw a hematologist. 

**Figure 2:**
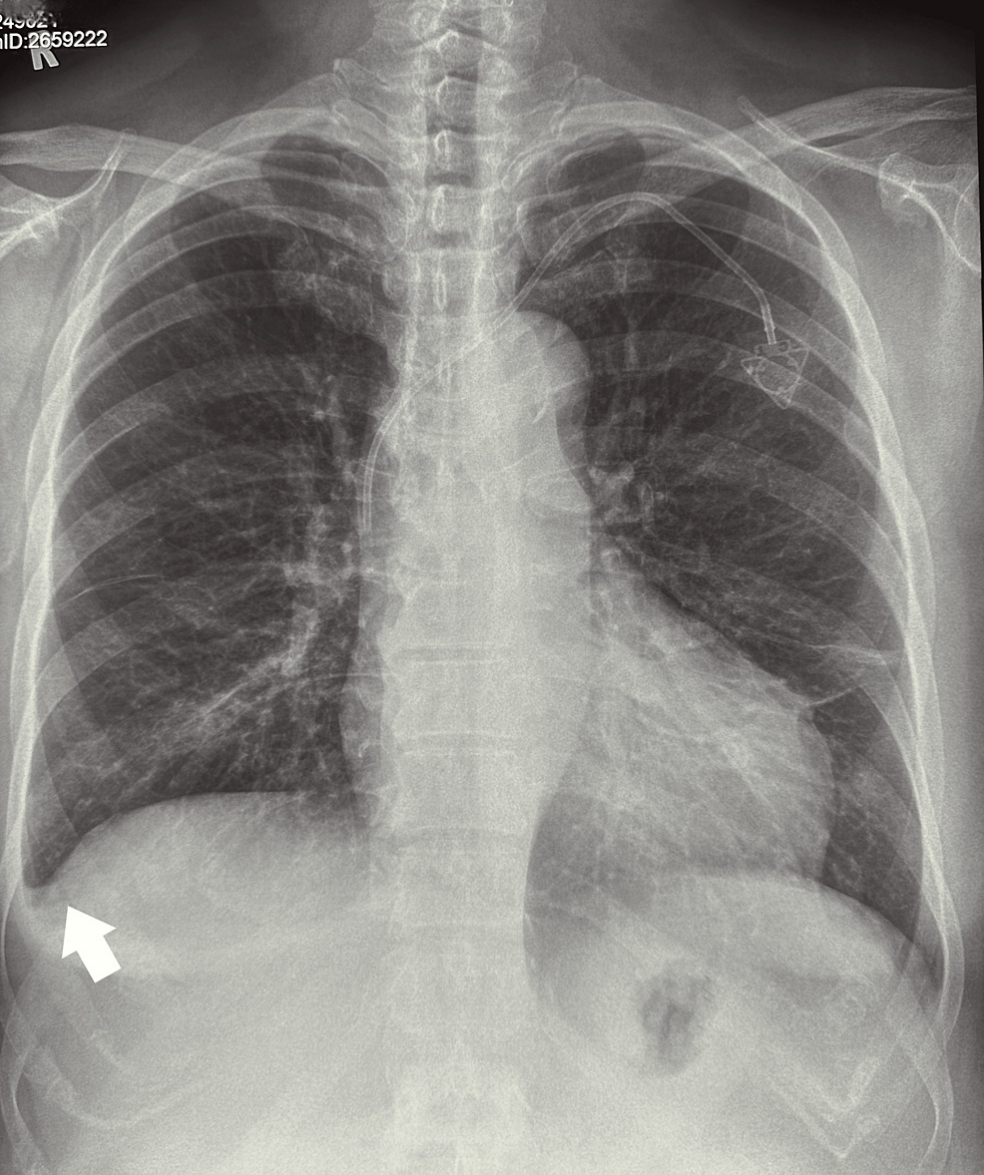
Chest radiograph Mild right lower zone infiltrates, linear density over the lateral left lower zone indicative of atelectasis, and blunting of the right costophrenic angle (white arrow) are indicative of pleural effusion. A chemo port is visible in the left side of the chest.

The hematologist performed a thorough physical examination. A peripheral blood smear revealed an increased number of lymphoblasts, suggestive of ALL. A bone marrow biopsy confirmed the presence of lymphoblasts, allowing a definitive diagnosis of ALL. Cytogenetic studies revealed a Philadelphia chromosome translocation, which helped determine a targeted treatment strategy using the tyrosine kinase inhibitor dasatinib. While in the hospital, the patient underwent a series of imaging studies, including chest, abdominal, and pelvic computed tomography. A chest radiograph revealed apparent blunting of the right costophrenic angle, which could indicate a small pleural effusion (Figure 2). The treatment plan was developed by a multidisciplinary team of specialists, including hematologists, oncologists, and pharmacists, and was divided into the following phases.

Targeted therapy with oral dasatinib (140 mg) and chemotherapy were initiated in the induction phase as the first-line treatment. During this phase, the patient’s severe anemia was treated with parenteral iron and blood transfusions. Upon achieving complete remission as evidenced by the disappearance of lymphoblasts from the bone marrow, the patient entered the consolidation phase of therapy. This phase involved intravenous chemotherapy over 24-hour periods for 28 days to eradicate any residual leukemia cells. The patient was closely monitored for signs of infection, and prophylactic antibiotics were administered as needed.

**Figure 3:**
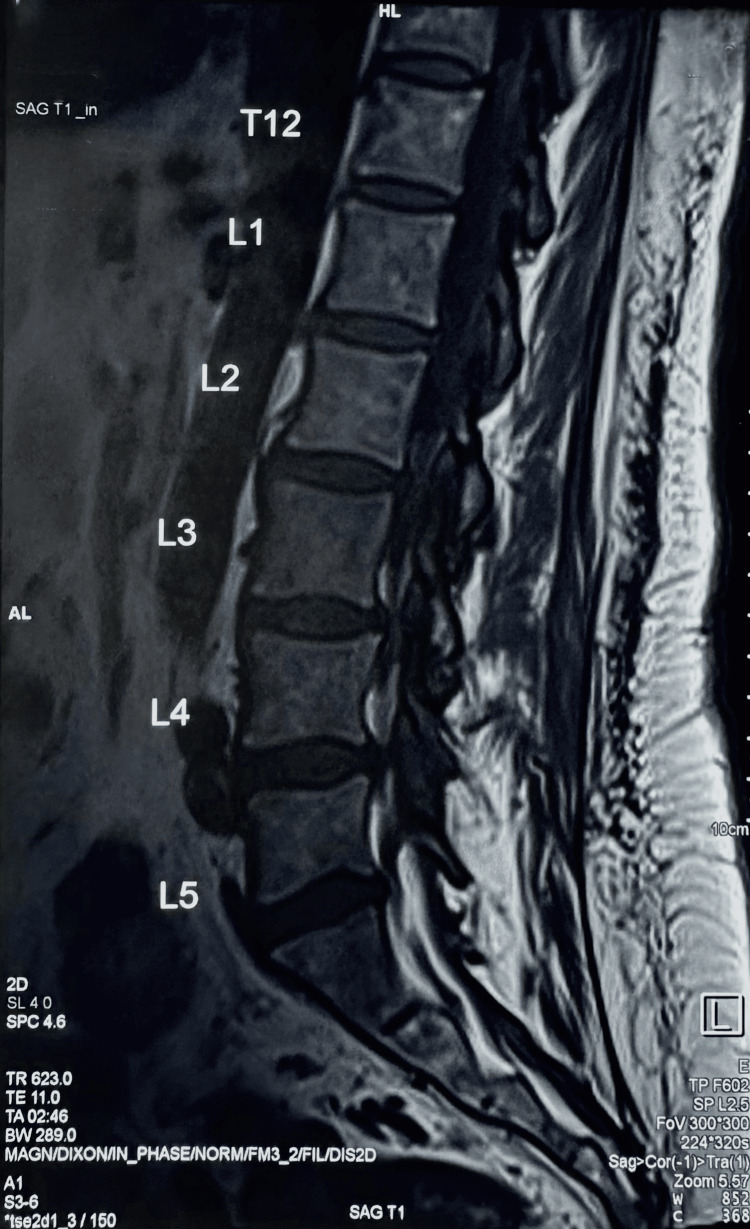
Lumbar MRI Lumbar MRI showing mild spondylotic changes of the lumbar spine. Features are most prominent at L3–4 with minimal narrowing of the spinal canal. No evidence of compression of the cauda equina was observed. MRI: Magnetic resonance imaging

Upon completion of the consolidation phase, the patient was transitioned to the maintenance phase of chemotherapy. Regular lumbar punctures were performed to assess CNS involvement and to allow the administration of intrathecal chemotherapy on alternating days as a preventative measure. All neurological examinations were negative, and the patient was discharged from the hospital in November 2022. However, bilateral numbness of lower extremities remained. Brain magnetic resonance imaging (MRI) was unremarkable; lumbar MRI showed tiny equivocal enhancing foci at the posterior aspect of the spinal canal at the L3 and L4 levels (Figure 3), with no evidence of compression of the cauda equina. Subsequently, the patient was diagnosed with herpes zoster, which was likely a complication secondary to a weakened immune system.

## Discussion

In this case report, we highlight the important role of chiropractors in identifying potential hematological disorders. Chiropractors often serve as primary points of contact for patients with musculoskeletal complaints, and their ability to recognize atypical presentations can facilitate early detection and treatment of underlying systemic conditions [13,14]. While the initial symptoms in the present case were suggestive of a musculoskeletal etiology, the patient's pale complexion, darkened facial features, and abnormal blood test results prompted a referral to a hematologist, ultimately leading to the timely diagnosis and treatment of ALL. Chiropractors' awareness of potential red flags in patient presentations can significantly impact the course of a patient's disease and their prognosis [15,16].

Differentiating between musculoskeletal and systemic causes of pain presents challenges for healthcare providers, as they can manifest similar symptoms [17-23]. In our case, the patient’s initial presentation of neck and back pain and lower extremity numbness and tenderness was consistent with a musculoskeletal origin. However, the persistence and severity of these symptoms, coupled with the patient's pale complexion and abnormal blood test results, raised the suspicion of a systemic cause. A thorough clinical evaluation, including a focused physical examination and appropriate laboratory investigations, is essential to distinguish between musculoskeletal and systemic causes and guide appropriate referrals and treatment plans.

In the present case, patient care involved a multidisciplinary team of specialists, including chiropractors, hematologists, oncologists, and radiation oncologists, who worked together to establish an accurate diagnosis and develop an effective treatment plan. This collaborative approach achieved complete remission of ALL with targeted and intrathecal chemotherapy. However, the patient's bilateral lower extremity paresthesia and numbness persisted because of herpes zoster neuralgia resulting from immunosuppression [24]. In immunosuppressed individuals, reduced T-cell immunity allows the dormant varicella-zoster virus in the dorsal root ganglia to reactivate, leading to viral spread within sensory neurons and the characteristic painful skin rash in the affected dermatomes [24]. Although antiviral therapy with famciclovir halts viral replication, nerve damage has already been sustained, manifesting as postherpetic neuralgia, which causes altered sensory processing and extreme pain that may persist for months or years [25].

Despite the novelty of the case presented here, it is important to acknowledge the limitations of individual case reports. Although case reports are valuable tools that can serve to highlight particular aspects of a disease or its treatment, they cannot establish causality or determine the prevalence of the condition. Nonetheless, this case serves as an important reminder for chiropractors and other healthcare providers to maintain a high index of suspicion for systemic causes of pain and highlights the importance of interdisciplinary collaboration in managing complex cases.

## Conclusions

This study highlights the crucial role of chiropractors as primary healthcare providers in identifying sinister hematopoietic pathologies underlying initial musculoskeletal symptomatology. In this case, expeditious recognition of red flags such as aberrant hematological indices and cutaneous pallor by the triaging chiropractic provider prompted swift interprofessional collaboration, allowing for the diagnosis of Philadelphia chromosome-positive B-cell ALL. Institution of targeted tyrosine kinase inhibition and serial intrathecal chemotherapy achieved complete cytogenetic remission. However, iatrogenic immunosuppression secondary to treatment may predispose patients to viral reactivation as evidenced by the development of herpetic neuralgia in this patient. Prudent history-taking, physical examination, and interpretation of laboratory test results are critical in determining the etiology underlying a patient’s symptoms and formulating an effective therapeutic strategy to achieve the best possible outcome. In this case, interprofessional cooperation facilitated a coordinated approach that effectively addressed the patient’s complex needs.
